# Association between occupational health literacy and occupational stress among workers in metal mining, metallurgy and non-metallic manufacturing in Gansu, China

**DOI:** 10.1186/s12889-025-25511-0

**Published:** 2025-11-25

**Authors:** Haiya Zhang, Wenli Zhao, Yuhong He, Jialong Wu

**Affiliations:** Department of Occupational Health, Gansu Center for Disease Control and Prevention, Lanzhou, 730000 China

**Keywords:** Occupational health literacy, Health literacy, Occupational stress, Mental health, Longer working hours

## Abstract

**Background:**

Occupational stress has become a significant public health concern, and health literacy (HL) is increasingly recognized as a potential influencing factor. However, most existing studies focus on knowledge-intensive occupational groups such as healthcare and education, while research on the relationship between occupational health literacy (OHL) and occupational stress in traditional industrial settings, such as factories and mines, remains limited.

**Methods:**

The study sample comprised 3,772 employees from the metal mining, metallurgy, and non-metallic mineral products industries located in Gansu Province. OHL was evaluated using the Individual Questionnaire of the National Key Population Occupational Health Literacy Monitoring Survey (IQ-NKPOHLMS), while occupational stress was measured via the Core Occupational Stress Scale (COSS). The analytical methods employed encompassed logistic regression modeling, restricted cubic spline (RCS) analyses, and subgroup analyses. These approaches were utilized to evaluate the association between occupational health literacy (OHL) and occupational stress, investigate potential dose-response relationships, and assess interaction effects.

**Results:**

Among participants, 55.6% exhibited adequate levels of occupational health literacy. Logistic regression analyses indicated that each one-point increment in OHL (continuous variable) was associated with a 2% reduction in the odds of experiencing occupational stress (OR = 0.981, 95% CI: 0.976–0.985). Furthermore, workers classified as having adequate OHL demonstrated a 31.6% lower likelihood of occupational stress compared to those with inadequate OHL (OR = 0.684, 95% CI: 0.589–0.793). The RCS analysis substantiated a significant linear dose-response relationship between OHL and occupational stress (*p* for nonlinearity = 0.2715). Subgroup analyses revealed that weekly working hours significantly moderated this association, with the protective effect of OHL being more pronounced among workers with shorter working hours; this interaction was statistically significant (*p* for interaction < 0.001).

**Conclusion:**

Adequate occupational health literacy is strongly linked to a lower risk of occupational stress, especially among employees who work fewer hours. This indicates that improving occupational health literacy should be a key part of workplace health initiatives, particularly for those involved in prolonged, demanding jobs. Additionally, strategies such as encouraging reasonable work hours, minimizing excessive overtime, and implementing focused occupational health literacy programs are advised to comprehensively enhance workers’ mental well-being.

**Supplementary Information:**

The online version contains supplementary material available at 10.1186/s12889-025-25511-0.

## Background

The rapid evolution of the global economy, coupled with technological advancements, has profoundly transformed work patterns across various occupational sectors. While these changes offer significant opportunities for societal progress and individual development, they also pose considerable challenges to workforce mental health [1]. Occupational stress has become a critical public health concern, attracting growing attention in both national and international research agendas in recent years [2, 3]. It is defined by adverse physical and emotional reactions that occur when job demands exceed an individual’s capabilities, resources, or needs [4]. Epidemiological studies have shown that prolonged exposure to occupational stress is linked to a range of physical and mental health disorders, including depression [5], anxiety [6], insomnia [7], musculoskeletal conditions [8], and cardiovascular diseases [9]. Moreover, individual factors such as educational attainment, income level, working hours, and night shift work have been identified as key determinants influencing vulnerability to occupational stress [10–13]. Health literacy (HL) has also emerged as a crucial factor deserving increased scholarly attention, with recent research suggesting its potential impact on workers’ experiences of occupational stress [14–17].

HL is defined as an individual’s ability to access, understand, evaluate, and apply health-related information effectively to engage in behaviors that promote health [18]. Over the past decade, as public health priorities have shifted from disease management to comprehensive health promotion, HL has gained prominence in academic discourse and has been incorporated into national health policies [19–21]. A strong association exists between HL and health outcomes; individuals with limited HL often exhibit reduced capacity to obtain and interpret health information, make informed health decisions, and adopt healthy behaviors, which may contribute to adverse health outcomes [19, 22–24].

Empirical evidence demonstrates a significant correlation between HL and both occupational performance and susceptibility to work-related illnesses [25, 26]. Furthermore, enhanced HL has been linked to improved mental health outcomes among employees [27, 28]. Research indicates a strong association between occupational stress and low levels of HL [17, 29]. It is important to note that existing studies primarily focus on knowledge-intensive occupational groups, such as healthcare workers and teachers, while relatively less attention has been given to workers in traditional industries like manufacturing and mining. Employees in these sectors generally have lower educational attainment and are more vulnerable to mental health problems due to high labor intensity, poor working conditions, repetitive and monotonous tasks, and exposure to occupational hazards such as dust and noise, particularly occupational stress [10, 30, 31]. For example, a national surveillance study of electronics manufacturing workers reported an occupational stress prevalence of 19.5% [32], whereas rates among coal mining workers range from 38% to 53% [33, 34]. These elevated prevalence rates contribute to a range of health complications and impose substantial economic burdens at societal, organizational, industrial, and individual levels. This underscores the critical need to identify determinants of occupational stress and implement targeted psychological interventions.Given that Gansu Province, a resource-rich region in western China, is home to a substantial workforce employed in factories and mines, addressing mental health concerns within this population is imperative.

Currently, HL is recognized as a strategic approach to enhancing health promotion and improving population health through better education and communication. Given the structured nature of organizational settings within the workforce, workplace-based health literacy interventions—particularly those tailored to specific occupational contexts—are likely to achieve greater acceptance and efficacy compared to general HL programs [35]. Consequently, occupational health literacy (OHL) assumes particular importance within the field of occupational health. To explore OHL and its effects within occupational populations, researchers have adapted general HL assessment instruments to develop occupation-specific tools [36, 37]. These studies integrate HL frameworks with occupation-specific variables such as work environments, behavioral patterns, and safety training, thereby providing valuable preliminary models for assessing OHL. Although HL assessment tools are diverse, most studies do not align the definition of HL with its measurement. Research on OHL is still in its early stages, and this issue persists [38].

To address this deficiency, the National Institute of Occupational Health and Poison Control at the Chinese Center for Disease Control and Prevention has defined OHL as workers’ awareness and ability to acquire essential occupational health knowledge, engage in health-promoting work behaviors, and effectively mitigate risks associated with occupational and work-related diseases, thereby promoting and protecting their health. Based on this definition and considering the unique characteristics of various occupations, the institute developed the “Individual Questionnaire of the National Key Population Occupational Health Literacy Monitoring Survey” (IQ-NKPOHLMS) [39]. This instrument covers four domains: knowledge of occupational health legislation, fundamental occupational health protection knowledge, core occupational health protection skills, and healthy working methods and behaviors [40]. This framework expands the conceptual scope of OHL, enabling a more comprehensive and accurate assessment of workers’ occupational health literacy.

In light of these challenges and future considerations, delineating the characteristics and determinants of occupational stress—particularly the role of OHL—among factory and mine workers is essential for informing public health strategies and governmental policies. This study employs the IQ-NKPOHLMS to assess the OHL levels of workers in three key industries in Gansu Province: metal mining, metallurgy, and non-metallic manufacturing. It also explores the interaction between OHL and occupational stress. The study posits the following hypotheses:

- Hypothesis 1 (H1): OHL has a negative effect on occupational stress.
- Hypothesis 2 (H2): Higher levels of OHL are associated with a lower risk of occupational stress after controlling for potential confounding factors.

## Method

### Study population

From May to December 2024, a stratified cluster random sampling method was employed to survey workers across three industries in Gansu Province: metal mining, metallurgy, and non-metallic mineral production. Enterprises were categorized according to the National Bureau of Statistics’ “Classification Method for Large, Middle, Small, and Micro Enterprises (2017)” based on the number of employees. Specifically, enterprises with fewer than 20 employees were classified as micro enterprises; those with 20–300 employees as small enterprises; those with 301–1000 employees as middle enterprises; and those with more than 1000 employees as large enterprises. Stratification was conducted by enterprise size, and 8% to 15% of enterprises were randomly selected from each stratum. The sampling design specified that large enterprises constitute 10% of the sample, while small and micro enterprises together account for 90%. The number of workers surveyed per enterprise was determined proportionally: 80–160 workers from large enterprises, 40–60 from medium enterprises, and 10–40 from small and micro enterprises. Ultimately, each industry included 1–2 large enterprises, 4–5 middle-sized enterprises, and 14–20 small or micro enterprises, resulting in a total of 71 enterprises from the three industries participating in the study. Detailed sampling information is provided in Supplementary Table 1.

**Fig. 1:**
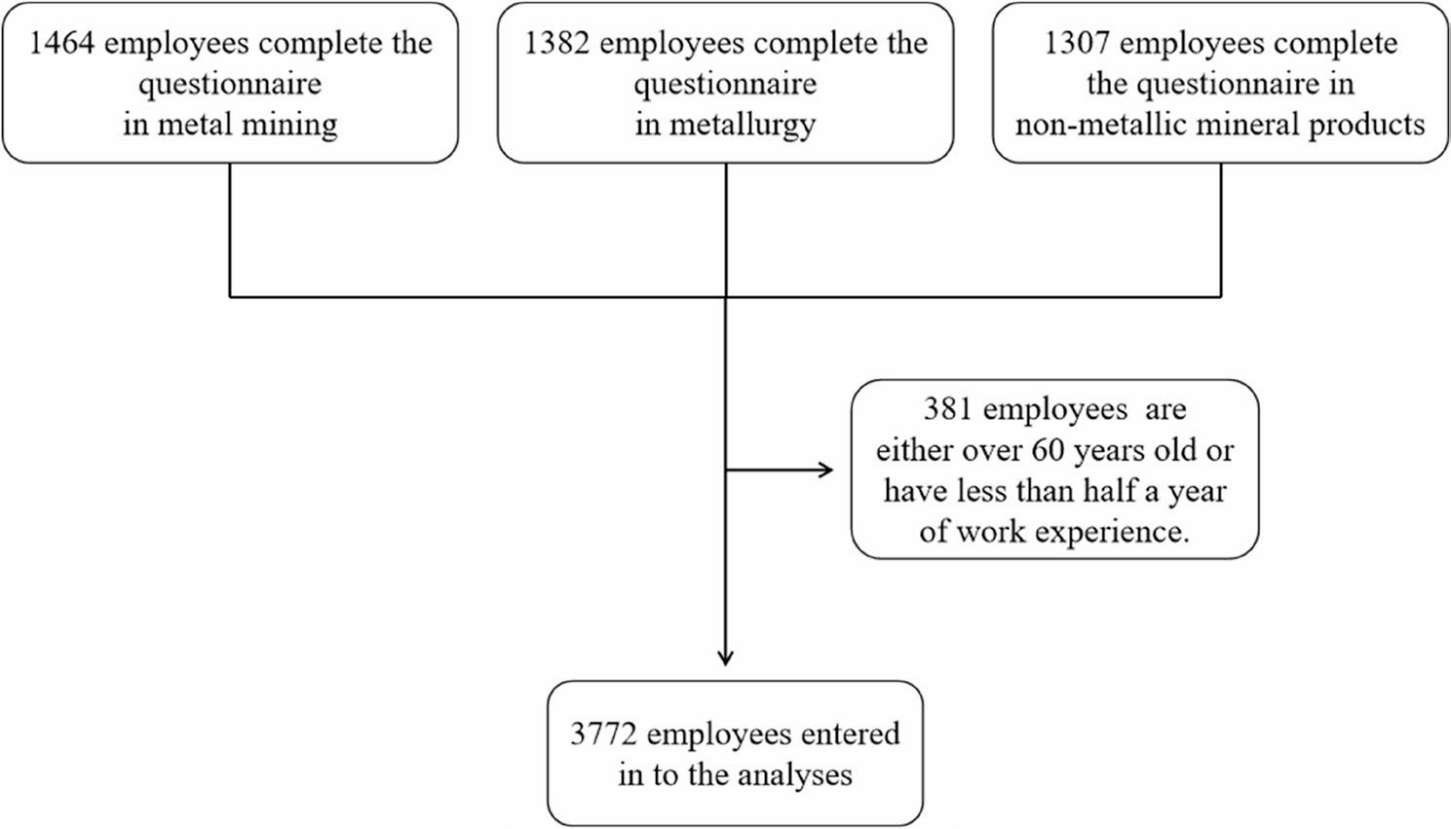
Flow chart of sample selection

Inclusion criteria for study participants required individuals to be aged between 16 and 59 years, possess work experience exceeding six months, and have no history of mental illness. The study received ethical approval from the Ethics Review Committee at the Gansu Provincial Center for Disease Control and Prevention (Ethics No.: 2024066), ensuring that all participants provided informed consent voluntarily. A total of 3,772 workers were included in this study, as depicted in Fig. 1.

### Assessment of occupational stress

Occupational stress was assessed through self-reporting using the “Core Occupational Stress Scale” (COSS), developed by the National Institute of Occupational Health and Poison Control at the Chinese Center for Disease Control and Prevention. This scale comprises four dimensions: social support (5 items), organization and rewards (6 items), demands and contributions (4 items), and autonomy (2 items), resulting in a total of 17 items. Each item is rated on a 5-point Likert scale, ranging from “strongly disagree” to “strongly agree.” The overall occupational stress score is calculated using the following formula: Total Occupational Stress Score = (6-B1) + (6-B2) + (6-B3) + (6-B4) + (6-B5) + B6 + B7 + B8 + B9 + B10 + B11 + B12 + B13 + B14 + B15 + (6-B16) + (6-B17). A cumulative score of 50 or higher indicates the presence of occupational stress. In this study, the Cronbach’s α coefficients for the overall scale and each dimension were found to be 0.776, 0.897, 0.802, 0.831, and 0.904, respectively, indicating strong reliability for the employed scale.

### Measurement of OHL

The assessment of OHL was conducted through the “Individual Questionnaire of the National Key Population Occupational Health Literacy Monitoring Survey” (IQ-NKPOHLMS). This evaluation encompasses four dimensions: knowledge of occupational health legislation (11 items), fundamental understanding of occupational health protection (14 items), essential skills for occupational health protection (4 items), and healthy work practices and behaviors (12 items), culminating in a total of 41 questions. The results derived from the questionnaire were analyzed in accordance with the “2022 Calculation Standards for Occupational Health Literacy Levels of Key Populations,” as established by the National Institute of Occupational Health and Poison Control at the Chinese Center for Disease Control and Prevention. The OHL score was calculated using the formula: OHL Score = (Total Correct Answers/Total Questions) * 100%. A score of 80.0% or higher is considered indicative of adequate OHL [40].

### Variables

The variables examined in this study included demographic and occupational information, which were gathered through a self-administered online questionnaire. Marital status was categorized into three groups: unmarried, married, and other. Educational attainment was classified as junior high school or below, high school, college, and undergraduate or higher. Monthly income (in Yuan) was segmented into categories: ≤3000, 3000–5000, 5000–7000, and ≥ 7000. Weekly work hours were classified as ≤ 40 h or > 40 h. Night work was defined dichotomously as “yes” or “no,” with “yes” indicating employment during the hours of 12:00 AM to 5:00 AM.

### Statistical analysis

Categorical variables were summarized using frequencies and percentages, and group differences were assessed using the chi-square test. Continuous variables were described using medians and interquartile ranges (IQR) based on their distributional characteristics, with group differences evaluated through nonparametric tests. Logistic regression model was employed to investigate the independent association between occupational stress and OHL. Three hierarchical regression models were developed: Model 1 served as the unadjusted basic model; Model 2 adjusted for demographic covariates (age, gender, ethnicity, marital status, education, household registration); and Model 3 further incorporated occupational-related factors (seniority, industry, enterprise size, income, work hours, night shifts). Restricted cubic splines (RCS) were utilized to explore potential non-linear relationships between continuous predictors and outcome variables. Subgroup analyses were conducted to assess the robustness and consistency of findings across various demographic subgroups, with results illustrated in forest plots. All statistical analyses were performed using SPSS version 26.0 and R version 4.4.2, with a significance threshold established at *p* < 0.05.

## Results

### Basic characteristics

The present study encompassed a sample of 3,772 employees, with a median age of 39.4 years (IQR = 17.0). The prevalence of occupational stress within this participants was identified as 30.7%. The majority of participants were male (80.4%) and of Han Chinese ethnicity (94.5%), with most being married (80.9%). The distribution between urban and rural residency was approximately equal (50.6% versus 49.4%). Educational attainment was generally low, as only 37.5% of the sample had completed higher education. Employees with 1 to 5 years of seniority represented the largest subgroup (34.2%), and nearly half were employed in micro enterprises (47.6%). The most frequently reported monthly income ranged from 3,001 to 5,000 yuan (44.8%). A considerable proportion of workers engaged in overtime and night work, accounting for 77.1% and 62.5%, respectively. The median OHL(continuous) was 81.8(IQR = 21.3), with more than half of the participants exhibiting adequate OHL levels. Statistically significant differences (*p* < 0.05) were observed between workers experiencing occupational stress and those without occupational stress across various sociodemographic and occupational variables, including gender, educational level, household registration status, seniority groups, industry sector, enterprise size, income, working hours, night work, and OHL (see Table 1).

**Table 1:** All characteristic of the participants (grouped by the occupational stress or without)

Characteristic	Overall	Occupational stress	Without Occupational stress	*p* Value
	*n* = 3772	*n* = 1157	*n* = 2615	
OHL(continuous)^a^	81.8(21.3)	78.9(22.8)	83.3(20.3)	**< 0.001**
OHL^b^				**< 0.001**
Inadequate	1673(44.4)	595(51.4)	1078(41.2)	
Adequate	2099(55.6)	562(48.6)	1537(58.8)	
Demography:				
Age^a^,years	39.4(17.0)	39.7(16.5)	39.2(17.1)	0.581
Age groups^b^,years				0.518
16 ~ < 30 years old	732(19.4)	210(18.2)	522(20.0)	
30 ~ < 40 years old	1217(32.3)	376(32.5)	841(32.2)	
40 ~ < 50 years old	1015(26.9)	325(28.1)	690(26.4)	
≥ 50 years old	808(21.4)	246(21.3)	562(21.5)	
Gender^b^				**< 0.001**
Male	3032(80.4)	982(84.9)	2050(78.4)	
Female	740(19.6)	175(15.1)	565(21.6)	
Ethnicity^b^				0.987
Han	3563(94.5)	1093(94.5)	2470(94.5)	
Minority	209(5.5)	64(5.5)	145(5.5)	
Marital status^b^				0.432
Unmarried	585(15.5)	168(14.5)	417(15.9)	
Married	3050(80.9)	943(81.5)	2107(80.6)	
Other	137(3.6)	46(4.0)	91(3.5)	
Education^b^				
Junior high school	1292(34.3)	403(34.8)	889(34.0)	**0.041**
High school	1064(28.2)	328(28.3)	736(28.1)	
College	920(24.4)	300(25.9)	620(23.7)	
Undergraduate	496(13.1)	126(10.9)	370(14.1)	
Household registration^b^				**0.006**
Urban	1908(50.6)	624(53.9)	1284(49.1)	
Rural	1864(49.4)	533(46.1)	1331(50.9)	
Occupational characteristics:				
Seniority^a^,years	5.1(11.0)	6.3(11.9)	4.8(10.7)	**< 0.001**
Seniority groups^b^, years				**< 0.001**
< 1	548(14.5)	127(11.0)	421(16.1)	
1 ~ < 5	1291(34.2)	381(32.9)	910(34.8)	
5 ~ < 10	655(17.4)	210(18.2)	445(17.0)	
10 ~ < 20	794(21.0)	268(23.2)	526(20.1)	
>=20	484(12.8)	171(14.8)	313(12.0)	
Industry^b^				**< 0.001**
Mining	1298(34.4)	342(29.6)	956(35.5)	
Metallurgy	1308(34.7)	410(35.4)	898(33.8)	
Non-metallic mineral products	1166(30.9)	405(35.0)	761(30.7)	
Enterprise size^b^				**< 0.001**
Micro	1797(47.6)	485(41.9)	1312(50.2)	
Middle	1260(33.4)	410(35.4)	850(32.5)	
Large	715(19.0)	262(22.6)	453(17.3)	
Income, yuan/month^b^				**0.002**
≤ 3000	514(13.6)	194(16.8)	320(12.2)	
3001 ~ < 5000	1690(44.8)	504(43.6)	1186(45.4)	
5001 ~ < 7000	1124(29.8)	334(28.9)	790(30.2)	
≥ 7000	444(11.8)	125(10.8)	319(12.2)	
Work time, hours/week^b^				**0.004**
≤ 40	862(22.9)	230(19.9)	632(24.2)	
> 40	2910(77.1)	927(80.1)	1983(75.8)	
Night work^b^				**< 0.001**
No	1415(37.5)	285(24.6)	1130(43.2)	
Yes	2357(62.5)	872(75.4)	1485(56.8)	

The significance of bold is *P* < 0.05

*Abbreviations*: *OHL* Occupational health literacy

^a^Median (IQR) is used to describe this variable, ^b^N (%) is used to describe this variable; P value reffects the difference between the two groups (occupational stress, without occupational stress)

### Association between OHL and occupational stress

A logistic regression analysis was performed to investigate the relationship between OHL and occupational stress, incorporating stepwise adjustments for potential confounding variables. OHL demonstrated a significant inverse association with occupational stress, regardless of whether it was treated as a continuous or categorical variable. Specifically, each one-point increment in OHL corresponded to a 2.0% reduction in the likelihood of experiencing occupational stress (OR = 0.980, 95% CI: 0.975–0.984). Following adjustments for demographic and occupational factors, the estimated risk reduction ranged from 2.2% to 1.9% (OR = 0.978, 95% CI: 0.973–0.983;OR = 0.981, 95% CI: 0.976–0.985). In the unadjusted model (Model 1), adequate OHL was significantly associated with decreased odds of occupational stress (OR = 0.662, 95% CI: 0.576–0.716). This association remained statistically significant after controlling for demographic variables (Model 2) and occupational variables (Model 3). Compared to individuals with inadequate OHL, those with adequate OHL exhibited a 34.7% to 31.6% lower risk of occupational stress (OR = 0.653, 95% CI: 0.568–0.752; OR = 0.684, 95% CI: 0.589–0.793, respectively) as detailed in Table 2.

**Table 2:** Associations between OHL and occupational stress (*N* = 3772)

	OHL(Continuous)	OHL	
		Inadequate	Adequate
	estimate(95%CI)		estimate(95%CI)
Occupational stress(OR)			
Model 1^a^	0.980(0.975,0.984)	reference	0.662(0.576,0.761)
Model 2^a^	0.978(0.973,0.983)	reference	0.653(0.568,0.752)
Model 3^a^	0.981(0.976,0.985)	reference	0.684(0.589,0.793)

Model 1 was unadjusted. Model 2 was adjusted for age, gender, ethnicity, marital status, education and household registration. Model 3 was adjusted for age, gender, ethnicity, marital status, education and household registration, seniority, industry, enterprise size, income, work time, night work

### Potential nonlinear relationship between OHL and occupational stress

**Fig. 2:**
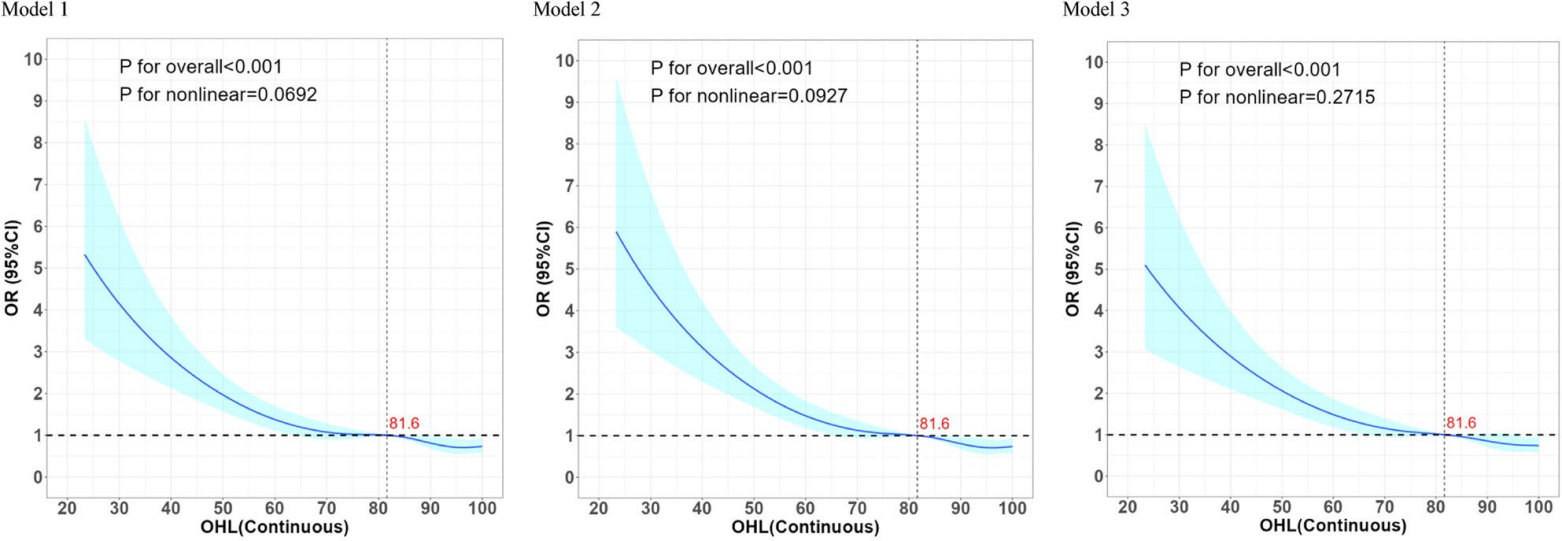
Restricted cubic spline for testing the hypothesis of non-linear correlation between Occupational health literacy(Continuous) and occupational stress. Note: Model 1 no adjusted; Model 2 adjusted age, gender, ethnicity, marital status, education, household registration; Model 3 adjusted age, gender, ethnicity, marital status, education and household registration, seniority, industry, enterprise size, income, work time, night work

RCS analyses did not indicate a significant nonlinear relationship between OHL and occupational stress. As shown in Fig. 2, Models 1, 2, and 3 consistently demonstrated a linear dose-response pattern: occupational stress declined sharply as OHL(Continuous) increased, followed by a gradual leveling off. The overall associations were statistically significant (Model 1: *p*_for overall_ < 0.001, *p*_for nonlinearity_ = 0.0692; Model 2: *p*_for overall_ < 0.001, *p*_for nonlinearity_ = 0.0927; Model 3: *p*_for overall_ < 0.001, *p*_for nonlinearity_ = 0.2715), with no strong evidence of curvature. Notably, a threshold effect was observed—protection against occupational stress improved markedly when OHL scores exceeded 81.6. Overall, adequate OHL were associated with a substantially lower risk of occupational stress (Fig. 2).

### Subgroup analysis

**Fig. 3:**
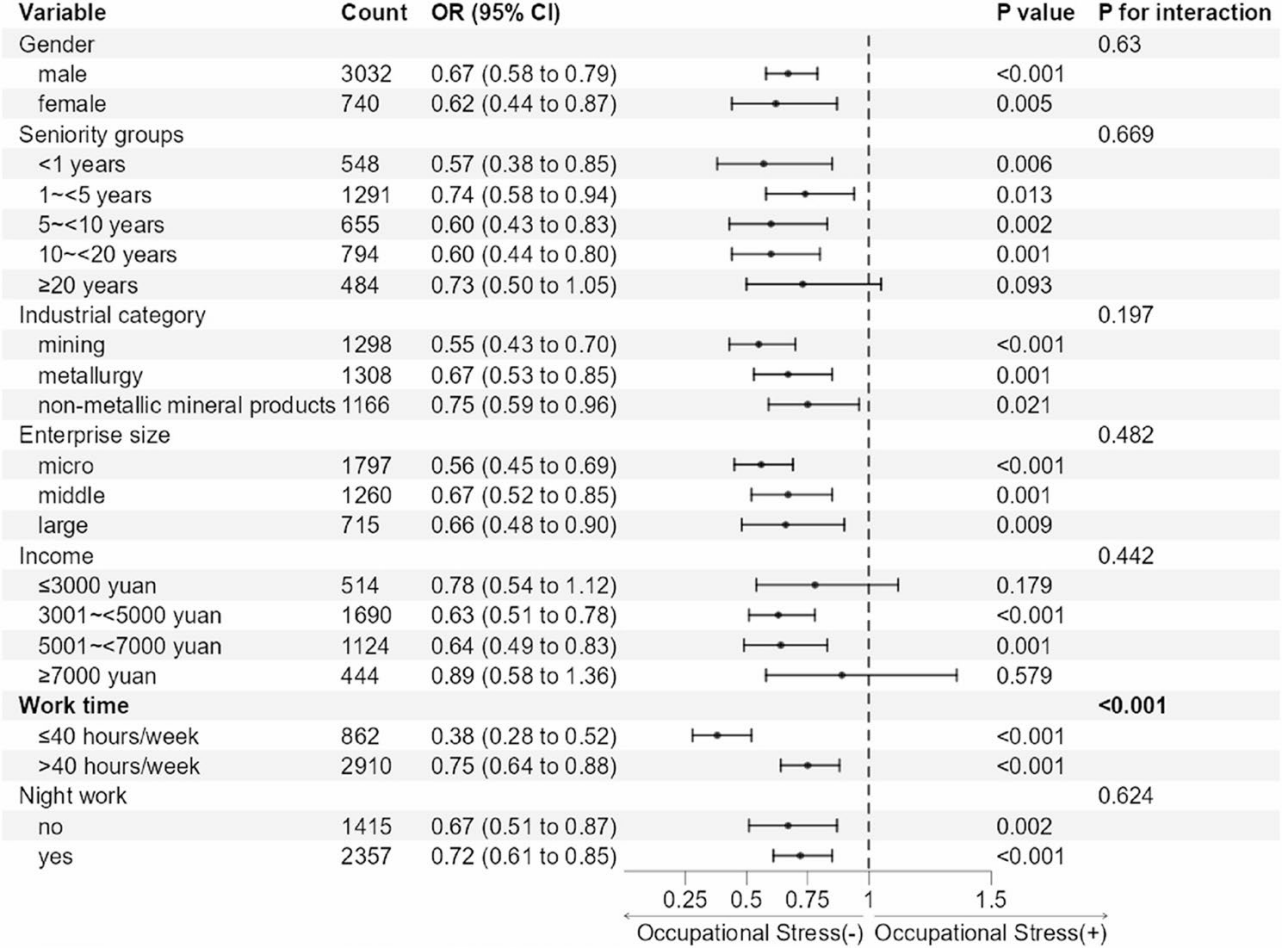
Interaction analysis of associations between OHL and occupational stress among different subgroups. Note: Adjusted for gender, industry, seniority groups, enterprise size, income, work time, night work

Subgroup analyses were conducted for variables that demonstrated statistical significance in the multivariable logistic regression model, including gender, seniority, industry, enterprise size, income level, working hours, and night shift work. As depicted in Fig. 3, adequate OHL was consistently associated with low odds of occupational stress across the majority of subgroups (*p* < 0.05). However, this association was not statistically significant among workers with 20 or more years of seniority and those earning monthly incomes of ≤ 3,000 yuan or ≥ 7,000 yuan. Furthermore, a significant interaction effect between weekly working hours and OHL was identified (*p* for interaction < 0.001). Specifically, the protective influence of adequate OHL against occupational stress was more pronounced in employees working 40 h or less per week compared to those working more than 40 h, as illustrated in Fig. 3.

## Discussion

We used the IQ-NKPOHLMS instrument to assess OHL and occupational stress levels among workers in three different industries. The results revealed a significant negative correlation between OHL and occupational stress: higher OHL levels were consistently associated with a lower risk of occupational stress. Furthermore, working hours significantly moderated this relationship, potentially enhancing the protective effect of OHL against occupational stress.

This study found that more than half (55.6%) of the participants exhibited adequate OHL levels. This proportion is lower than that reported among port operators but higher than in other occupational groups [41–43]. These differences may be attributed to two factors: first, variations in regional economic conditions, cultural backgrounds, and individual characteristics across study populations influence OHL levels; second, as an emerging area within HL research, OHL currently lacks standardized and unified measurement tools, leading to the use of diverse assessment scales across studies and limiting direct comparability of findings.

The results corroborate Hypothesis 1, demonstrating a significant negative correlation between OHL and occupational stress, consistent with previous studies [17, 29, 41]. Empirical evidence suggests that individuals possessing higher HL are more inclined to adopt beneficial health behaviors, such as taking timely rest when fatigued, appropriately adjusting work pace, maintaining a balance between work and personal life, and regularly monitoring their health status [15]. These behaviors collectively contribute to mitigating psychological strain and enhancing mental well-being, thereby diminishing the likelihood of experiencing occupational stress. Adequate HL thus serves a protective function against occupational stress [29]. Workers in industrial and mining settings encounter complex work environments characterized by high-intensity tasks alongside various occupational health hazards, which constitute the primary sources of their occupational stress. Prior research predominantly concentrated on general HL, employing assessment instruments that primarily measured health knowledge [17, 29]. In contrast, the present study’s evaluation of OHL is congruent with its conceptual definition, utilizing a questionnaire that comprehensively addresses occupational health-related knowledge, encompassing both fundamental and legal aspects. Consequently, the HL assessed herein is more specifically tailored and relevant to occupational contexts. Our findings indicate that adequate OHL is associated with a 31.6% reduction in the risk of occupational stress, underscoring the substantial protective effect of adequate OHL on the occupational stress experienced by workers in industrial and mining enterprises.

At the personal level, various factors have been associated with occupational stress [10–12, 29]. Our results indicate that male workers, those with longer job seniority, lower income, and night shift workers face a higher risk of occupational stress—findings largely consistent with previous studies. Additionally, we observed variations in occupational stress risk across industries and enterprise sizes, likely due to differences in production environments, management systems, and labor intensity. Importantly, subgroup analyses revealed that adequate OHL was consistently associated with a reduced risk of occupational stress across all demographic and occupational subgroups, indicating that the protective effect of adequate OHL is robust and mostly unaffected by major variables. This finding supports Hypothesis 2.

However, the moderating effect of working hours varied significantly. Subgroup analysis revealed a significant interaction between OHL and weekly working hours. Specifically, workers with sufficient OHL who worked 40 h or less per week had a notably lower risk of experiencing occupational stress compared to those working more than 40 h. This suggests that maintaining standard working hours provides greater protection against occupational stress. Previous research has identified long working hours as a major risk factor for occupational stress [11, 32, 44, 45]. Data indicate that working over 40 h per week increases the risk of occupational stress by 90%, and this risk escalates to 170% when working beyond 60 h [44]. Extended working hours not only reduce personal and leisure time but can also heighten psychological stress through several mechanisms: excessive overtime may decrease sleep duration, cause sleep deprivation, activate the hypothalamic-pituitary-adrenal (HPA) axis, and provoke stress responses [45]. Additionally, consistently high work intensity limits opportunities for learning and skill development, restricting individuals’ ability to gain and apply OHL knowledge. Therefore, the moderating effect of working hours on the relationship between OHL and occupational stress likely operates through these indirect pathways. This finding underscores the importance of working hours as a contextual factor influencing the protective effects of OHL against occupational stress, providing new understanding of how work conditions and health literacy interact dynamically.

This study has several limitations. First, its cross-sectional design prevents establishing a causal link between occupational health literacy (OHL) and occupational stress. Second, data were mainly gathered through self-administered questionnaires, which could be affected by participants’ education levels, understanding, and other personal factors, possibly leading to reporting bias. Third, although previous research shows that workplace hazards like asbestos, benzene, and noise significantly contribute to occupational stress [10, 31], this study did not measure actual exposure to such hazards (e.g., dust, noise) due to logistical challenges, limiting our ability to account for their potential confounding effects. Lastly, since the sample was taken from Gansu Province, an area with unique economic and cultural traits, the findings may have limited applicability to other regions.

## Conclusion

The role of HL in promoting mental health is increasingly recognized. This study provides cross-sectional yet valuable evidence on the association between OHL and occupational stress. We recommend integrating OHL into core components of workplace health promotion programs and enhancing workers’ capacity to access, understand, and apply health information through structured training initiatives, thereby effectively mitigating occupational stress. Additionally, greater attention should be directed toward the mental health of workers with long working hours. Targeted OHL interventions should be implemented for this high-risk group, alongside efforts to promote reasonable working hour policies and reduce excessive overtime, to holistically improve mental health.

## Supplementary Information

Supplementary Material 1.

Supplementary Material 2.

## Data Availability

The corresponding author can provide the data supporting the study’s conclusions upon request.

## Abbreviations

HL: Health literacy
OHL: Occupational health literacy
COSS: Core occupational stress scale
IQ-NKPOHLMS: National key population occupational health literacy monitoring survey
IQR: Interquartile ranges
RCS: Restricted cubic splines
