# Supplementary material for: Association between occupational health literacy and occupational stress among workers in metal mining, metallurgy and non-metallic manufacturing in Gansu, China
Source: BMC Public Health. 2025 Nov 25;25:4320. doi: 10.1186/s12889-025-25511-0 (PMC12751191; doi:10.1186/s12889-025-25511-0)
Supplement: Supplementary file 1 — Supplementary Material 1. [file 12889_2025_25511_MOESM1_ESM.docx]

Supplementary Table 1 Distribution of Enterprises and Sampling in metal mining, metallurgy and non-metallic manufacturing in Gansu,China

| Industry | Enterprise size | Number of enterprises | Number of sampled enterprises (%) | Total number of employees in sampled enterprises | Number of sampled employees（%） |
| --- | --- | --- | --- | --- | --- |
| metal mining | large | 7 | 2（9.52） | 3089 | 335（10.84） |
|  | middle | 23 | 5（23.81） | 2488 | 308（12.37） |
|  | micro | 109 | 14（66.67） | 1199 | 821（68.47） |
|  | Subtotal | 139 | 21（100） | 6776 | 1464（21.61） |
| metallurgy | large | 20 | 2（8.7） | 4177 | 270（6.46） |
|  | middle | 28 | 6（26.09） | 1798 | 504（28.03） |
|  | micro | 92 | 15（65.22） | 957 | 608（63.53） |
|  | Subtotal | 140 | 23（100） | 6932 | 1382（19.94） |
| non-metallic mineral production | large | 2 | 2（7.41） | 1785 | 251（14.06） |
|  | middle | 24 | 5（18.52） | 835 | 583（69.82） |
|  | micro | 637 | 20（74.07） | 494 | 473（95.74） |
|  | Subtotal | 663 | 27（100） | 3114 | 1307（41.97） |
| Total |  | 942 | 71（100） | 16822 | 4153（24.69） |
