# Supplementary material for: Association between occupational health literacy and occupational stress among workers in metal mining, metallurgy and non-metallic manufacturing in Gansu, China
Source: BMC Public Health. 2025 Nov 25;25:4320. doi: 10.1186/s12889-025-25511-0 (PMC12751191; doi:10.1186/s12889-025-25511-0)
Supplement: Supplementary file 2 — Supplementary Material 2. [file 12889_2025_25511_MOESM2_ESM.pdf]

Supplementary Table 1 Distribution of Enterprises and Sampling in metal mining, metallurgy and non-metallic manufacturing in Gansu,China

Industry	Enterprise size	Number of enterprises	Number of sampled enterprises (%)	Total number of employees in sampled enterprises	Number of sampled employees（%）
metal mining	large	7	2（9.52）	3089	335（10.84）
	middle	23	5（23.81）	2488	308（12.37）
	micro	109	14（66.67）	1199	821（68.47）
	Subtotal	139	21（100）	6776	1464（21.61）
metallurgy	large	20	2（8.7）	4177	270（6.46）
	middle	28	6（26.09）	1798	504（28.03）
	micro	92	15（65.22）	957	608（63.53）
	Subtotal	140	23（100）	6932	1382（19.94）
non-metallic mineral production	large	2	2（7.41）	1785	251（14.06）
	middle	24	5（18.52）	835	583（69.82）
	micro	637	20（74.07）	494	473（95.74）
	Subtotal	663	27（100）	3114	1307（41.97）
Total		942	71（100）	16822	4153（24.69）

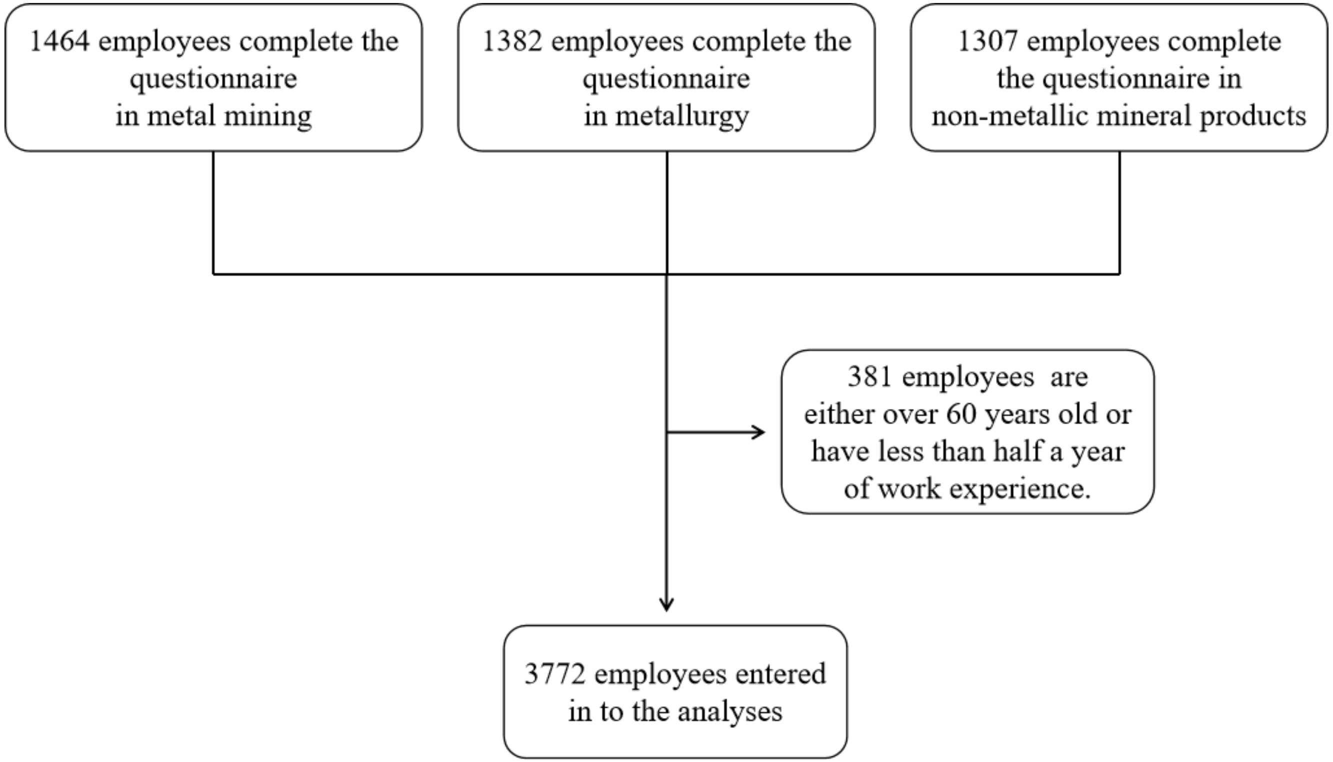

Fig. 1 flow chart of sample selection

Table1.All characteristic of the particpants(grouped by the occupational stress or without)

Characteristic	Overall	Occupational stress	Without Occupational stress	*p* Value
	n=3772	n=1157	n=2615	
OHL(continuous)[a]	81.8(21.3)	78.9(22.8)	83.3(20.3)	**<0.001**
OHL[b]				**<0.001**
Inadequate	1673（44.4）	595（51.4）	1078（41.2）	
Adequate	2099（55.6）	562（48.6）	1537（58.8）	
Demography:				
Age[a],years	39.4(17.0)	39.7(16.5)	39.2(17.1)	0.581
Age groups[b],years				0.518
16~<30 years old	732(19.4)	210(18.2)	522(20.0)	
30~<40 years old	1217(32.3)	376(32.5)	841(32.2)	
40~<50 years old	1015(26.9)	325(28.1)	690(26.4)	
≥50 years old	808(21.4)	246(21.3)	562(21.5)	
Gender[b]				**<0.001**
male	3032(80.4)	982(84.9)	2050(78.4)	
female	740(19.6)	175(15.1)	565(21.6)	
Ethnicity[b]				0.987
han	3563(94.5)	1093(94.5)	2470(94.5)	
minority	209(5.5)	64(5.5)	145(5.5)	
Marital status[b]				0.432
unmarried	585(15.5)	168(14.5)	417(15.9)	
married	3050(80.9)	943(81.5)	2107(80.6)	
other	137(3.6)	46(4.0)	91(3.5)	
Education[b]				
junior high school	1292(34.3)	403(34.8)	889(34.0)	**0.041**
high school	1064(28.2)	328(28.3)	736(28.1)	
college	920(24.4)	300(25.9)	620(23.7)	
undergraduate	496(13.1)	126(10.9)	370(14.1)	
Household registration[b]				**0.006**
urban	1908(50.6)	624(53.9)	1284(49.1)	
rural	1864(49.4)	533(46.1)	1331(50.9)	
Occupational characteristics:				
Seniority[a],years	5.1(11.0)	6.3(11.9)	4.8(10.7)	**<0.001**
Seniority groups[b],years				**<0.001**
<1	548(14.5)	127(11.0)	421(16.1)	
1~<5	1291(34.2)	381(32.9)	910(34.8)	
5~<10	655(17.4)	210(18.2)	445(17.0)	
10~<20	794(21.0)	268(23.2)	526(20.1)	
>=20	484(12.8)	171(14.8)	313(12.0)	
Industry[b]				**<0.001**
mining	1298(34.4)	342(29.6)	956(35.5)	
metallurgy	1308(34.7)	410(35.4)	898(33.8)	

non-metallic mineral products	1166(30.9)	405(35.0)	761(30.7)	
Enterprise size[b]				**<0.001**
micro	1797(47.6)	485(41.9)	1312(50.2)	
middle	1260(33.4)	410(35.4)	850(32.5)	
large	715(19.0)	262(22.6)	453(17.3)	
Income,yuan/month[b]				**0.002**
≤3000	514(13.6)	194(16.8)	320(12.2)	
3001~<5000	1690(44.8)	504(43.6)	1186(45.4)	
5001~<7000	1124(29.8)	334(28.9)	790(30.2)	
≥7000	444(11.8)	125(10.8)	319(12.2)	
Work time,hours/week[b]				**0.004**
≤40	862(22.9)	230(19.9)	632(24.2)	
>40	2910(77.1)	927(80.1)	1983(75.8)	
Night work[b]				**<0.001**
no	1415(37.5)	285(24.6)	1130(43.2)	
yes	2357(62.5)	872(75.4)	1485(56.8)	

Abbreviations: OHL, Occupational health literacy

The signiffcance of bold is $P < 0.05$

Note:a:median (IQR) is used to describe this variable;b:N (%) is used to describe this variable; P value reffects the difference between the two groups (occupational stress, Without occupational stress)

Table2.Associations between OHL and occupational stress (N = 3772)

	OHL(Continuous)	OHL	
		Inadequate	Adequate
	estimate(95%CI)		estimate(95%CI)
occupational stress(OR)			
Model 1[a]	0.980(0.975,0.984)	reference	0.662(0.576,0.761)
Model 2[a]	0.978(0.973,0.983)	reference	0.653(0.568,0.752)
Model 3[a]	0.981(0.976,0.985)	reference	0.684(0.589,0.793)

Note:Model 1 was unadjusted. Model 2 was adjusted for age, gender, ethnicity, marital status, education and household registration. Model 3 was adjusted for age, gender, ethnicity, marital status, education and household registration, seniority, industry, enterprise size, income, work time, night work.

Model 1 Model 2 Model 3

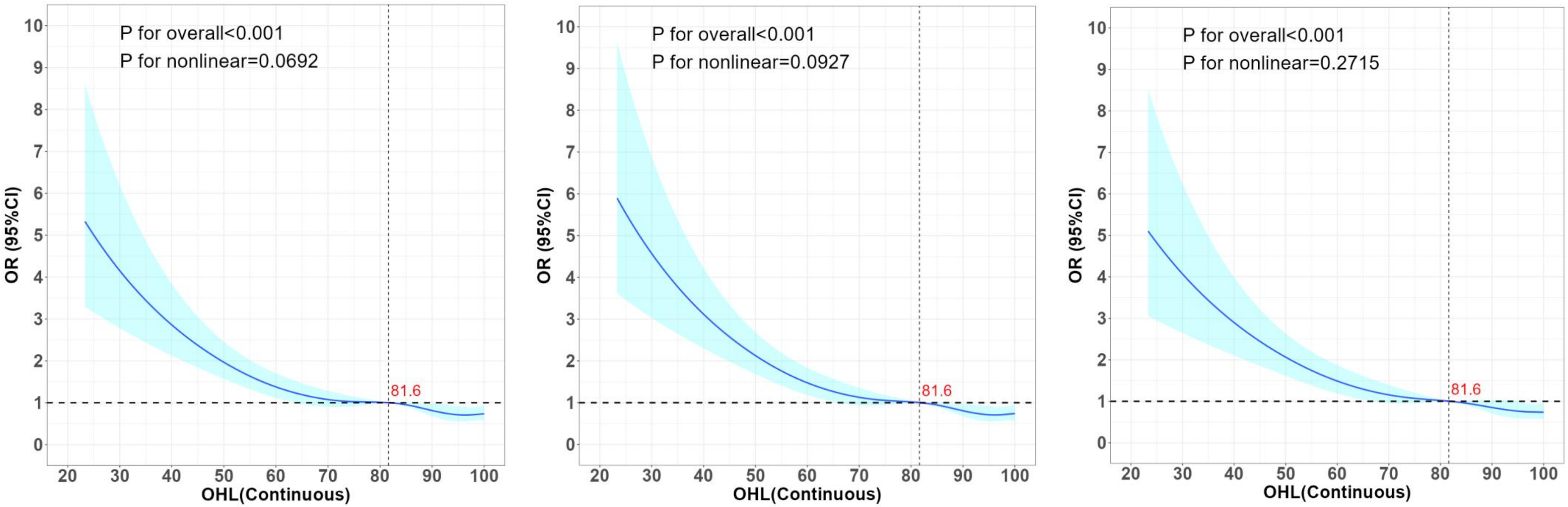

Fig.2 Restricted cubic spline for testing the hypothesis of non-linear correlation between Occupational health literacy(Continuous) and occupational stress.

Note:Model 1 no adjusted; Model 2 adjusted age, gender, ethnicity, marital status, education, household registration;Model 3 adjusted age, gender, ethnicity, marital status, education and household registration, seniority, industry, enterprise size, income, work time, night work;

Variable	Count	OR (95% CI)	P value	P for interaction
Gender				0.63
male	3032	0.67 (0.58 to 0.79)	<0.001	
female	740	0.62 (0.44 to 0.87)	0.005	
Seniority groups				0.669
<1 years	548	0.57 (0.38 to 0.85)	0.006	
1~<5 years	1291	0.74 (0.58 to 0.94)	0.013	
5~<10 years	655	0.60 (0.43 to 0.83)	0.002	
10~<20 years	794	0.60 (0.44 to 0.80)	0.001	
≥20 years	484	0.73 (0.50 to 1.05)	0.093	
Industrial category				0.197
mining	1298	0.55 (0.43 to 0.70)	<0.001	
metallurgy	1308	0.67 (0.53 to 0.85)	0.001	
non-metallic mineral products	1166	0.75 (0.59 to 0.96)	0.021	
Enterprise size				0.482
micro	1797	0.56 (0.45 to 0.69)	<0.001	
middle	1260	0.67 (0.52 to 0.85)	0.001	
large	715	0.66 (0.48 to 0.90)	0.009	
Income				0.442
≤3000 yuan	514	0.78 (0.54 to 1.12)	0.179	
3001~<5000 yuan	1690	0.63 (0.51 to 0.78)	<0.001	
5001~<7000 yuan	1124	0.64 (0.49 to 0.83)	0.001	
≥7000 yuan	444	0.89 (0.58 to 1.36)	0.579	
**Work time**				**<0.001**
≤40 hours/week	862	0.38 (0.28 to 0.52)	<0.001	
>40 hours/week	2910	0.75 (0.64 to 0.88)	<0.001	
Night work				0.624
no	1415	0.67 (0.51 to 0.87)	0.002	
yes	2357	0.72 (0.61 to 0.85)	<0.001	

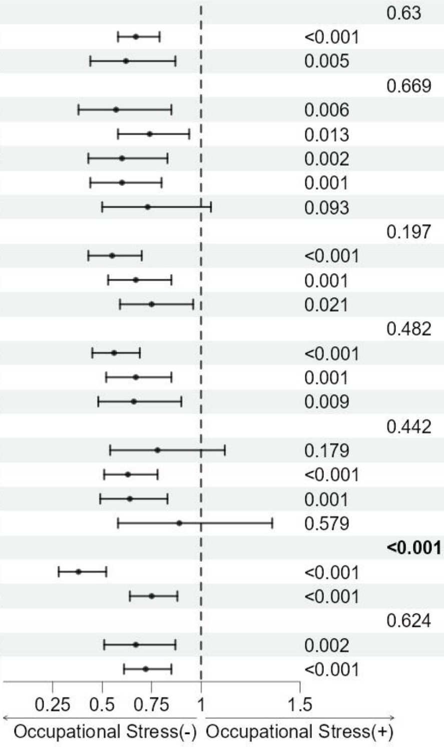

Fig. 3 Interaction analysis of associations between OHL and occupational stress among different subgroups.

Note: Adjusted for gender, industry, seniority groups, enterprise size, income, work time, nigh twork.
